# Comparison of analgesic efficacy between intrafunicular and intratesticular lidocaine injection in dogs

**DOI:** 10.3389/fvets.2026.1753113

**Published:** 2026-01-22

**Authors:** Christian Lohinger, Natali Verdier, Moriz Ettore Klonner, Christina Braun, Ulrike Auer

**Affiliations:** Unit of Anaesthesiology and Intensive Care, Clinical Department for Small Animals and Horses, University of Veterinary Medicine Vienna, Vienna, Austria

**Keywords:** dogs, intrafunicular, intratesticular, lidocaine, orchiectomy, regional anaesthesia, analgesia, cortisol

## Abstract

Elective surgical orchiectomy is a commonly performed procedure in dogs that requires adequate pain management. Multiple studies have shown that various local anaesthetic techniques improve analgesia and may reduce side effects associated with systemically administered drugs. This study aimed to compare the analgesic efficacy of an injection and an ultrasound-guided intrafunicular injection of lidocaine. In total, 28 dogs were included in this study: 13 dogs received an intratesticular injection, whereas 15 dogs received an ultrasound-guided intrafunicular injection. Assessed data included the time needed to perform the locoregional technique, time between locoregional anaesthesia and skin incision, removal of the second testicle, and end of surgery; intraoperative vital parameters, quality of recovery, postoperative pain scores, and serum cortisol levels. The time to perform the ultrasound-guided intrafunicular block (96.13 ± 30.67 s) was significantly longer than the intratesticular block (23.20 ± 5.57 s). Both techniques proved to be feasible and effective with a similar success rate.

## 1 Introduction

Elective surgical orchiectomy is a commonly performed procedure in dogs (1). To provide safe and effective analgesic and anaesthetic management, the procedure is commonly performed under general anaesthesia, and a multimodal analgesic approach is used (2). Whilst perioperative analgesia can be provided using systemically administered drugs, locoregional anaesthesia interrupts the transmission of nociceptive stimuli and, therefore, plays a key role in perioperative pain management (3). In light of these benefits, the use of locoregional anaesthesia has been recommended in all procedures whenever possible, considering its high efficacy and low incidence of adverse effects (4).

Several locoregional anaesthetic blocks have been proposed for dogs undergoing orchiectomy. Intratesticular (IT) or intrafunicular (IF) injections of local anaesthetics, with and without incisional line infiltration, as well as epidural anaesthesia, have been described, showing decreased intra- and postoperative nociception when compared with a traditional systemic analgesic approach (1, 5–10). Interestingly, the analgesia provided by an IT injection of lidocaine was shown to be comparable to that of an epidural injection of morphine in dogs (11). The IT locoregional anaesthesia has been extensively investigated and compared to various other locoregional or systemic analgesic approaches. An ultrasound-guided IF (UGIF) block has been investigated, albeit to a lesser extent, showing promising results for effective postoperative analgesia (6). Additionally, the IF injection of local anaesthetics may be convenient in cases where an IT injection is not recommended, such as testicular neoplasia or scrotal dermatitis.

In comparison to control groups, in which no local blocks were performed, an IT as well as a UGIF have been proven to offer superior intra- and postoperative analgesic efficacy. The analgesic efficacy was evaluated by intraoperative vital parameters and postoperative pain scores. However, a comparison of the analgesic efficacy between an IT and a UGIF block in dogs is lacking (1, 6).

The stress response is defined as hormonal and metabolic changes following injury or trauma. It is part of a systemic reaction to injury that includes multiple haematological, endocrinological, and immunological effects. Serum cortisol levels have been used as indicators to measure the stress response. Cortisol secretion from the adrenal cortex increases rapidly following surgical intervention as a result of stimulation by ACTH (3, 12, 13). In dogs, it has been shown that general anaesthesia alone without any surgical treatment had no significant change in plasma cortisol levels. In contrast, surgical procedures under general anaesthesia increased plasma cortisol levels significantly (14). Furthermore, cortisol has been widely used as a parameter to quantify the stress response and assess the analgesic efficacy of regional anaesthetic blocks in dogs (14–17).

This study aimed to (1) assess the feasibility of a UGIF block in anaesthetised dogs undergoing elective orchiectomy and (2) to compare its intraoperative and immediate postoperative analgesic efficacy with the traditional IT block by assessing intraoperative vital parameters, serum cortisol levels, and postoperative pain scores. It was hypothesised that the UGIF block provides comparable or better intraoperative and immediate postoperative analgesia in dogs undergoing elective orchiectomy.

## 2 Materials and methods

The study was approved by the Ethics Committee of the University of Veterinary Medicine, Vienna, in accordance with the University’s guidelines for Good Scientific Practice, and authorized by the Austrian Federal Ministry of Education, Science and Research [ref BMBWF (2020-0.282.309)]. Informed owner’s written consent was obtained prior to surgery for all animals enrolled in the study.

### 2.1 Animals

Sample size determination was based on a study performed in humans, in which 20 patients received an ultrasound-guided intrafascial block to test the feasibility and monitor the success rate of an ultrasound-guided spermatic cord block (18). According to that, a convenience sample of 40 dogs was selected. Client-owned male dogs, scheduled to undergo elective surgical castration, were assessed for eligibility. Inclusion criteria were healthy dogs based on clinical examination, normal haematology and serum biochemistry, absence of testicular pathology, an American Society of Anaesthesiologists physical status of I, and animals in which anaesthetic premedication with acepromazine and methadone intramuscularly (IM) was considered suitable. Dogs were excluded if they presented testicular pathology (e.g., neoplasia, scrotal dermatitis, cryptorchidism), systemic disease, in which a premedication with acepromazine and methadone was not considered suitable (e.g., aggressive behaviour and ABCB1 mutation), or if they were scheduled to undergo an additional surgical procedure during the same anaesthetic event.

### 2.2 Anaesthetic management

Food, but not water, was withheld for 12 h prior to anaesthesia. All dogs were administered 0.02 mg/kg acepromazine (Vanastress®, VANA GesmbH, Austria) and 0.2 mg/kg methadone (Methadone Streuli AG, Switzerland) intramuscularly (IM) as preanaesthetic medication. After 30 min, an intravenous catheter (Vasofix® Safety, B. Braun Austria GmbH, Austria) was placed in the cephalic vein. A balanced crystalloid solution (Sterofundin® ISO 1/1 E Iso, B. Braun Austria GmbH, Austria) was administered at a rate of 5 mL/kg/h intravenously (IV) throughout the procedure (19). Following mask preoxygenation for 3 min with 100% oxygen and a flow rate of 100 mL/kg/min, general anaesthesia was induced with propofol (Propofol 1%, Fresenius Kabi, Austria) IV titrated to effect until orotracheal intubation with a cuffed and appropriately sized endotracheal tube (RÜSCH Super Safetyclear RÜSCH Austria GmbH, Austria) was achieved. The endotracheal tube was tied, cuffed, and attached to a circle breathing system. General anaesthesia was maintained with isoflurane (Vetflurane, Virbac Animal Health Netherlands, Netherlands) in 100% oxygen, using an end-tidal concentration (EtIso%) of 1.3–1.6%. Dogs were allowed to breathe spontaneously. All dogs were administered 0.2 mg/kg meloxicam (Metacam Injektionsloesung fuer Hunde und Katzen, Boehringer Ingelheim Vetmedica GmbH, Germany) IV after induction. Anaesthetic depth was assessed by means of the palpebral reflex, eye globe position, and jaw muscle tone. Electrocardiogram (ECG), heart rate (HR), respiratory rate (RR), pulse oximetry (SpO_2_), end-tidal carbon dioxide (EtCO_2_), oesophageal temperature, and oscillometric non-invasive blood pressure (systolic, mean and diastolic blood pressure) by means of an appropriately sized cuff placed on one hind leg were monitored continuously using a multiparameter monitor (Philips Intellivue MP60, Philips Medical Systems, Germany) and recorded every 5 min.

### 2.3 Locoregional anaesthesia

All dogs received a locoregional anaesthetic block before surgery. Dogs were randomly assigned to one of two groups: an IT (Group IT) or a UGIF (Group IF) injection of local anaesthetic. Randomisation was performed by drawing opaque envelopes. Dogs were placed in dorsal recumbency, and the surgical area was aseptically prepared. The time needed (in seconds) for the locoregional anaesthetic block, from the beginning of manipulation or ultrasound scanning to the end of injection, was recorded. Locoregional anaesthesia was performed by either one of the two authors (CL; MEK).

#### 2.3.1 IT injection

Group IT was administered a total amount of 3 mg/kg lidocaine 2% (xylocaine 2% Ampulle Injektionsloesung, Aspen Germany GmbH, Germany), i.e., 1.5 mg/kg per testicle using a 23 G hypodermic needle (TERUMO® AGANI™ Needle, 0.6 × 32 mm, Shanghai International Holding Corp. GmbH, Germany). Briefly, the testicle was manually fixed at the cranial and caudal poles with the thumb and index finger of the non-dominant hand. Then, the hypodermic needle was inserted through the scrotum into the testicle, aiming at the estimated centre of the testicle, and 1.5 mg/kg of lidocaine was administered after negative aspiration whilst the needle was slowly withdrawn. The injection was repeated in the contralateral testicle.

#### 2.3.2 UGIF injection

**Figure 1:**
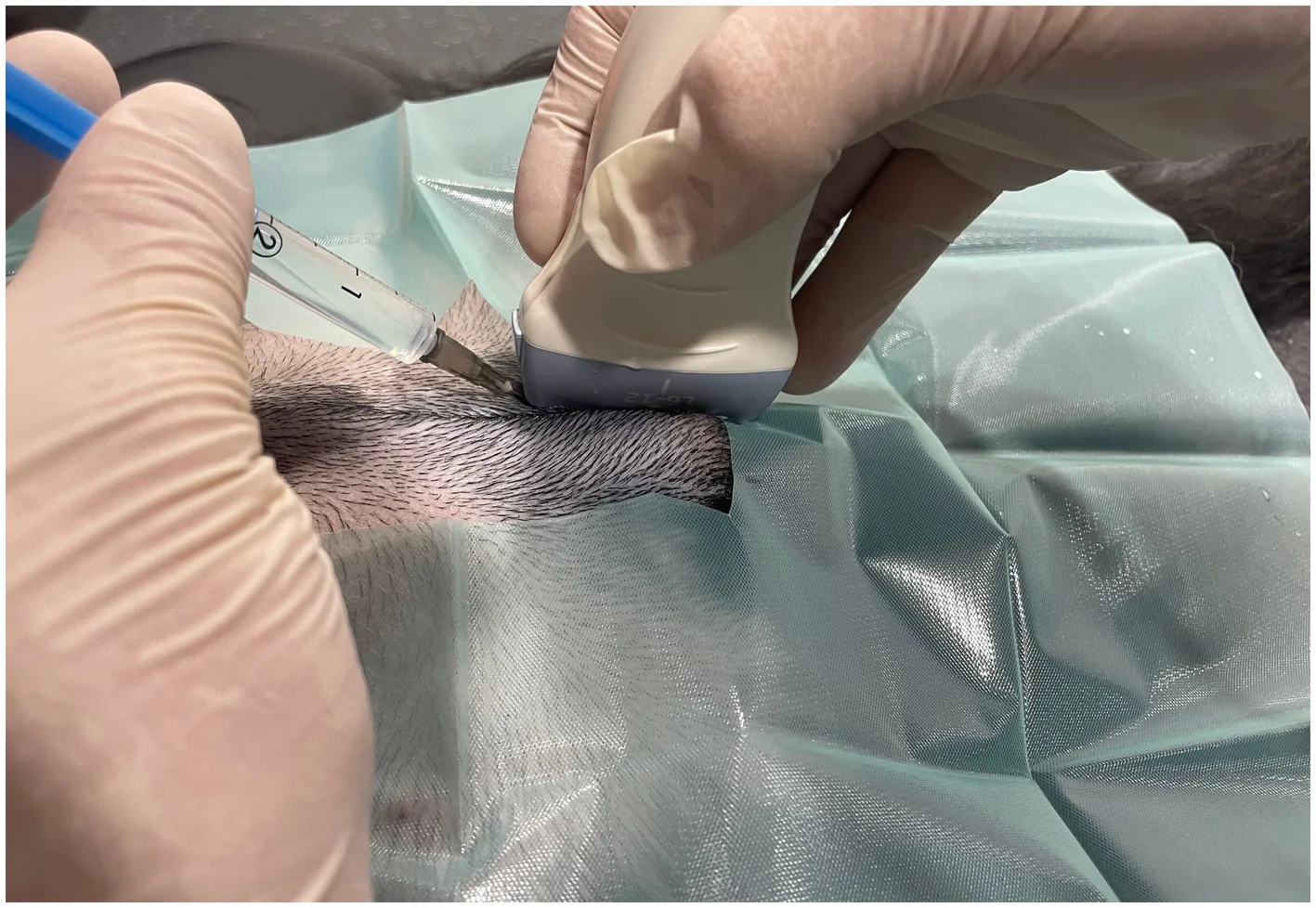
Representation of the positioning of the ultrasound transducer and the insertion of the needle for performing a UGIF block in dogs (N.B. the ultrasound transducer used for this study and for the image in Figure 2 is of 5–10 MHz).

**Figure 2:**
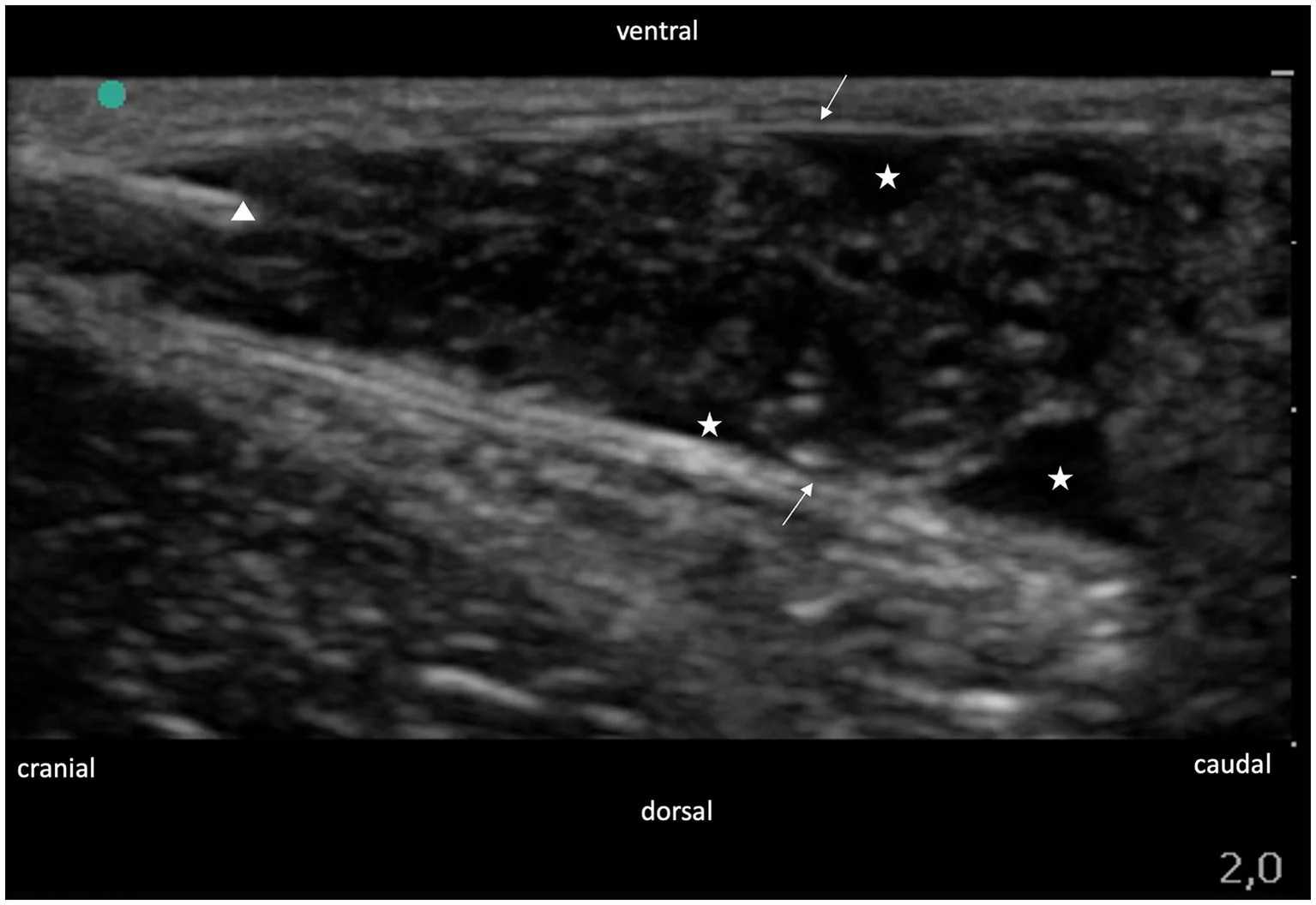
Ultrasonographic image for the visualisation of the injection site for the UGIF block. Arrowhead: tip of the hypodermic needle; stars: local anaesthetic that has already been injected; between arrows: spermatic cord.

Group IF was administered a total amount of 3 mg/kg lidocaine 2% (xylocaine 2% Ampulle Injektionsloesung, Aspen Germany GmbH, Germany), i.e., 1.5 mg/kg per spermatic cord under ultrasound guidance using a 23 G hypodermic needle (TERUMO® AGANI™ Needle, 0.6 × 32 mm, Shanghai International Holding Corp. GmbH, Germany). A 38 mm broadband linear array transducer (L38n/10–5 MHz) attached to an ultrasound unit (SonoSite® NanoMaxx, FUJIFILM Sonosite Europe, Netherlands) was used to visualise the spermatic cord. The ultrasound probe was placed prescrotally in a craniocaudal direction. After visualisation of the spermatic cord, the needle was advanced in a craniocaudal direction until its tip was located within the spermatic cord (Figures 1, 2). Subsequently, 1.5 mg/kg of lidocaine 2% was administered after negative aspiration. The injection was repeated in the contralateral testicle.

### 2.4 Intraoperative regime

All animals were transferred to the operating theatre. Baseline heart rate (HR) and mean arterial blood pressure (MAP) values were obtained over 10 min prior to surgical incision, every 3 min by taking the average of three consecutive readings. After starting surgery, these variables were then measured every 5 min throughout the procedure. An increase in HR and/or MAP 20% above baseline was interpreted as a nociceptive response, and a bolus of 0,001 mg/kg IV fentanyl (Fentanyl-Janssen®, Janssen-Cilag Pharma GmbH, Austria) was administered (20, 21). The bolus was repeated every 5 min until HR and MAP were restored to baseline values or values within 20% increase from baseline. The amount of intraoperative fentanyl administered was recorded for each animal.

Intraoperative anaesthetic complications were treated using atropine 0.04 mg/kg IV in case of bradycardia and two IV boluses (3 mL/kg over 3 min.) if hypotension occured. If treatment for hypotension was still required, dogs were administered stepwise increases in dopamine (5–15 μg/kg/min). The management decision was based on the anaesthetist’s discretion.

Surgery was performed by a specialist at the clinical center of animal reproduction of the same institution.

### 2.5 Serum cortisol testing

Serum cortisol levels (μg/dL) were determined by sampling 0.5 mL of peripheral venous blood at the following time-points: catheter placement (T1, baseline), clamping of first testicle (T2), clamping of second testicle (T3), 30 min after extubation (T4), and 120 min after extubation (T5). The blood sample for T1 was obtained immediately after placing the venous catheter used for the induction of general anaesthesia. The other samples were obtained from an additional 20 G intravenous catheter placed in the contralateral cephalic vein after induction and connection to the anaesthesia machine. All samples were collected into a sample tube with a serum clot activator (Vacuette® Serumroehrchen Gerinnungsaktivator, Greiner Bio-One GmbH, Kremsmünster, Austria) and were analysed using chemiluminescent assay (IMMULITE 2000 XPi©, Siemens Healthcare Diagnostics GmbH, Austria) by the Clinical Pathology Unit of the same institution.

### 2.6 Postoperative management

Following completion of surgery, dogs were transferred to the recovery area for further assessment and care. Upon return of the swallowing reflex, the trachea was extubated, and the time was noted. Quality of recovery was assessed over a 30-min period after tracheal extubation, using a score presented in Table 1 adapted from another study (22). Postoperative pain was evaluated at 30, 60, 90, and 120 min after tracheal extubation, using the short form of the Glasgow Composite Pain Scale (SF-GCPS) (23). In case of an SF-GCPS score ≥ 5, methadone 0.2 mg/kg IV was administered as a rescue analgesia. Postoperative pain scores were assessed by the anaesthetist in charge of the patient. The time from extubation to the first methadone administration was recorded for all dogs. All dogs were discharged on the day of surgery.

**Table 1:** Descriptive recovery score used to assess the quality of recovery of all dogs 30 min after extubation [adjusted from Larenza et al.(22)].

Recovery score	Description
0	Normal behavioural patterns (quiet, grooming, attentive)
1	Minor behavioural changes (minor incoordination and excitation)
2	Moderate behavioural changes (moderate incoordination and excitation)
3	Severe behavioural changes (rolling over, severe incoordination, and excitation)

### 2.7 Statistical analysis

The distribution of all continuous variables was tested using the Shapiro–Wilk test. For normally distributed data, a mixed-model ANOVA (two-way repeated measures ANOVA) was used to assess the effects of group (between-subjects factor), time (within-subjects factor), and their interaction (group × time) on pain scores, cortisol levels, vital parameters, and analysed time points. Post-hoc pairwise comparisons were performed using Bonferroni correction. For non-normally distributed data, the Friedman test was applied within each group to assess changes over time, and Mann–Whitney U-tests were used to compare groups at each time point, with a Bonferroni correction for multiple comparisons. Statistical significance was considered with a *p*-value of < 0.05. Statistical analyses were performed using NCSS 2023, version 23.0.2.

## 3 Results

A total of 28 male dogs were enrolled. No dog was excluded from this study. They were 34.11 ± 25.45 months of age and weighed 15.95 ± 10.75 kilograms. A total of 13 dogs were allocated to the IT group and 15 dogs to the IF group. There were no significant differences in age and weight between the groups. The propofol dosage needed for induction was 4.07 ± 1.35 mg/kg.

No complications occurred during block execution in any dog from both groups. The spermatic cord, as well as the hypodermic needle used for injection, could be identified ultrasonographically in all dogs in the IF group.

The time needed to perform the block in IT was 23.20 ± 5.57 s, and it was significantly lower (*p* ≤ 0.001) than in IF (96.13 ± 30.67 s). The time interval between the administration of locoregional anaesthesia and the skin incision was 17.08 ± 6.97 min for the IT group and 15.60 ± 8.58 min for the IF group (*p* = 0.6196). The time between skin incision and the removal of the second testicle was 29.31 ± 13.20 min and 28.20 ± 12.67 min, respectively (*p* = 0.8227). The time between locoregional anaesthesia and the end of surgery for IT was 40.95 ± 16.73 min and 38.20 ± 13.05 min (*p* = 0.6489), respectively.

During surgery, no significant differences in vital parameters could be detected between the two groups (Table 2). One dog in the IT group needed one single fentanyl bolus at the time of skin incision, whereas one dog in the IF group needed one single bolus at the time of skin closure.

**Table 2:** Intraoperative vital parameters.

Variable	Group	Timepoint					
		Skin incision	5	10	15	20	End of surgery
HR (beats per minute)	IT	80 (56–138)	79 (57–116)	89 (70–120)	93 (69–110)	89 (62–116)	84 (59–115)
	IF	88 (53–139)	89 (66–109)	83 (72–116)	82 (69–107)	79 (69–106)	73 (41–99)
	*p*	0.5645	0.3447	0.4331	0.9700	0.1225	0.0615
EtCO_2_ (mmHg)	IT	43 (18–52)	44 (23–52)	43 (20–51)	45 (23–52)	44 (22–51)	43 (22–50)
	IF	46 (36–55)	45 (39–55)	45 (39–57)	45 (38–57)	45 (38–55)	47 (35–55)
SpO_2_ (%)	IT	100 (99–100)	100 (99–100)	100 (97–100)	100 (97–100)	100 (97–100)	100 (97–100)
	IF	100 (99–100)	100 (99–100)	100 (98–100)	100 (99–100)	100 (98–100)	100 (99–100)
RR (breaths per minute)	IT	12 (4–28)	8 (4–27)	9 (4–22)	9 (4–24)	10 (3–24)	11 (4–23)
	IF	15 (6–31)	11 (6–18)	11 (5–18)	10 (4–18)	10 (4–20)	10 (5–20)
	*p*	0.5790	0.4050	0.6112	0.9815	0.7987	0.5581
SAP (mmHg)	IT	109 (88–146)	110 (92–146)	120 (84–146)	106 (83–156)	115 (82–148)	122 (84–135)
	IF	112 (89–210)	101 (87–151)	103 (80–159)	110 (81–187)	109 (87–194)	104 (81–178)
	*p*	0.9265	0.5186	0.5185	0.8718	0.8537	0.1280
MAP (mmHg)	IT	69 (60–99)	68 (59–105)	69 (61–106)	69 (61–115)	71 (61–111)	79 (59–106)
	IF	70 (61–145)	70 (59–100)	64 (61–119)	72 (60–150)	70 (59–141)	67 (60–135)
	*p*	0.9265	0.7465	0.9082	0.5798	0.5491	0.3687
DAP (mmHg)	IT	45 (28–78)	45 (29–87)	45 (27–86)	50 (29–90)	52 (35–92)	56 (32–87)
	IF	51 (33–108)	54 (40–107)	50 (36–79)	55 (34–96)	55 (33–87)	50 (34–98)

HR, heart rate; EtCO_2_, end-tidal CO_2_; SpO_2_, oxygen saturation; RR, respiratory rate; SAP, systolic arterial blood pressure; MAP, mean arterial blood pressure; DAP, diastolic arterial blood pressure in dogs receiving an ultrasound-guided intrafunicular (IF) or an intratesticular (IT) injection of 2% lidocaine at all time points starting from skin incision, every 5 min until 25 min (last timepoint) or until end (if later than 20 min but earlier than 25 min). Data are expressed as median (range). The *p*-values represent differences between groups at a given timepoint.

Postoperative pain scores were not significantly different between groups at any time point. No animals needed methadone as rescue analgesia during this period (Table 3).

**Table 3:** Postoperative pain scores according to the Short Form of the Glasgow Composite Pain Scale in dogs receiving an ultrasound-guided intrafunicular (IF) or an intratesticular (IT) injection of 2% lidocaine at time points 30, 60, 90, and 120 min after tracheal extubation.

Group	30 min	60 min	90 min	120 min
IF	1.60 (±1.12)	1.47 (±0.82)	1.27 (±0.88)	0.93 (±0.88)
IT	1.85 (±1.34)	1.23 (±0.93)	1.38 (±1.61)	1.00 (±1.08)
*p*	0.6070	0.4896	0.8166	0.8588

Data are expressed as mean (± SD). The *p*-values represent differences between groups at a given timepoint.

In both groups, serum cortisol levels were higher at the first measurement (baseline) than at the subsequent timepoints, and higher than the laboratory reference range (0.50–3.20 μg/dL) at T1 and T3 for IF.

Serum cortisol levels were not significantly different between groups at any timepoint (Table 4).

**Table 4:** Serum cortisol levels (μg/dL) in dogs receiving an ultrasound-guided intrafunicular (IF) or an intratesticular (IT) injection of 2% lidocaine at time points: T1: catheter placement, T2: clamping first testicle, T3: clamping second testicle, T4: 30 min after extubation, and T5: 120 min after extubation.

Group	T1	T2	T3	T4	T5
IT	2.88 (± 1.48)	1.78 (± 0.95)	2.26 (± 1.30)	1.93 (± 1.11)	2.20 (± 1.56)
IF	4.30 (± 2.58)	2.95 (± 2.08)	3.65 (± 2.81)	2.28 (± 1.11)	1.98 (± 1.13)
*p*	0.0764	0.0569	0.1866	0.4232	0.6584

Data are expressed as mean (± SD). The *p*-values represent differences between groups at each timepoint.

## 4 Discussion

This study demonstrated that the UGIF injection of 2% lidocaine was feasible and resulted in comparable perioperative analgesia and serum cortisol levels to the traditional IT block. The time needed to perform the IT block was significantly shorter than the time needed to perform the UGIF injection.

### 4.1 Feasibility of the UGIF block

The UGIF block using a hypodermic needle was feasible in all the included dogs, which confirms the findings by Cicirelli et al. (6). Although the referenced study used a Quincke type 22G, 0.7 × 90 mm spinal needle, the differences in length and echogenicity between the needles used in this study did not hinder the execution of the block, and in both cases, the block was deemed feasible. Although the block in this study was performed by operators experienced in ultrasound-guided regional anaesthesia, the technique was subjectively easy to perform, which was in agreement with the above-referenced study. In general, the training of ultrasound-guided locoregional anaesthetic techniques has a long learning curve, but the identification of relevant structures and injection targets for this block was possible and straightforward. Whilst the time required to perform the UGIF block was longer compared to the IT block, the longest time required was 150 s, which does not seem to be a clinically relevant duration. Nevertheless, it is noteworthy that, conversely to the IT block, the UGIF block requires additional and expensive equipment, namely a high-frequency ultrasound transducer as well as an ultrasound unit. In all dogs in this study, the UGIF block occurred without complications, which is also in agreement with Cicirelli et al. (6). There were no adverse effects, neither those associated with the technique nor those associated with the injection of local anaesthetics. The advancement of the needle under ultrasound guidance and the identification of the target structure allow for a reduction in the volume of injectate needed to achieve adequate nerve desensitisation and therefore reduce the risk of local anaesthetic systemic toxicity (24). Although in this study different volumes of injectate were not evaluated, the UGIF as performed did not result in any complications in the dogs in this study.

### 4.2 Perioperative analgesic efficacy of IT versus IF

#### 4.2.1 Intraoperative period

One dog in each group needed one fentanyl bolus as rescue analgesia. The dog in the IT group received fentanyl at the time of skin incision, whereas the dog allocated to IF required fentanyl during skin closure. It was assumed that fentanyl was necessary in both groups due to a lack of skin desensitisation. Skin infiltration has been recommended to provide desensitisation of the incision line (7). However, in the current study, skin infiltration with local anaesthetic of the incision area was not performed to avoid a possible confounding factor when comparing the two techniques. Based on these results, both techniques were equally effective in providing desensitisation of the testicles, which is in agreement with the available literature. One study showed that when a UGIF was performed, no rescue analgesia was needed intraoperatively (6). Similarly, it has also been shown that the use of an IT block results in a lower requirement for systemic analgesic and anaesthetic drugs (7, 8). Another case report demonstrated no significant differences between an IT and an IF injection of local anaesthetics regarding the consumption of rescue analgesia (25). Further studies with a larger sample size are warranted to characterise the spread of local anaesthetic, the extent of the desensitised area, and the analgesic efficacy of both techniques, with and without skin desensitisation.

#### 4.2.2 Postoperative period

Pain scores were not significantly different between groups at any time point, indicating a similar analgesic efficacy provided by the two techniques in this study. Pain sores showed a tendency to decrease in both groups from first to last assessment, which might also be due to the effect of meloxicam administration (Table 3). These observations are in agreement with other studies in dogs receiving a UGIF (6) or an IT (9) block for orchiectomy, for which no rescue analgesia was needed postoperatively (6, 9).

#### 4.2.3 Serum cortisol levels

In both groups, serum cortisol levels showed a tendency to decrease along the measured timepoints and were above the reference range at T1 and T3 for the IF group. Between groups, cortisol levels per timepoint were not significantly different. Interestingly, a slight increase was noted in both groups between timepoints T2 and T3, i.e., between clamping of the first and second testicles. During the increase in serum cortisol, no simultaneous nociceptive response in any patient of any group could be detected. Studies measuring cortisol during surgical interventions describe increases in cortisol levels as early as 15 min after painful stimuli or even only after extubation (26–29), and therefore, it could be that cortisol levels in response to the clamping of the first testicle were evident later. However, the lack of nociceptive responses during T2 and T3, and the overall decreasing tendency of cortisol levels in both groups, led us to assume that the slight increase in cortisol at T3 was not associated with a nociceptive stimulus. Other factors that may influence cortisol levels have been described, such as the fasting period, individual patterns, breed, physical activity, sleep, rest during day and night (26, 30), and noise stimuli (31). It has been shown that dogs that fasted for 12 to 24 h have lower cortisol levels than non-fasted dogs (32). In this study, all owners were instructed to withhold food the night before surgery, so differences based on fasting were assumed unlikely. It has also been shown that cortisol levels can increase significantly 6 min after noise stimuli and remain elevated for up to 15 min (31); however, such events were not recorded in this study, and all dogs were exposed to the same clinical conditions. The possible influence of all these factors has not been evaluated in this study, but the homogeneous characteristics of the included dogs and the similar cortisol trend let us assume that both locoregional techniques were effective in desensitisation of the testicles.

### 4.3 Time between injection and surgery

Despite the different durations for performing the IF and the IT block, there was no significant difference in time between the skin incision and the removal of the second testicle. As no significant difference in vital parameters between groups and within groups was observed, we could assume that the spread of lidocaine after 30 min was sufficient to provide testicular desensitisation. A study in piglets showed that IT injection of radioactive lidocaine reached the highest concentration of lidocaine in the spermatic cord 3 min after injection, and decreased by approximately 90% until 40 min after injection (33). Another study in horses, in which radioactive lidocaine was administered intratesticularly, revealed diffuse distribution within the spermatic cord at the time of removal of the testicles, 12.3 min in one stallion and 12.7 min in another stallion (34). Autoradiograms in both studies also revealed poor distribution into the cremaster muscle, and nociceptive responses and twitches in the cremaster muscle were noticed, which indicated that desensitisation was not complete. It would be optimal to perform orchiectomy when the concentration of local anaesthetic in the spermatic cord is the highest; however, to the authors’ knowledge, no reports exist on the distribution of local anaesthetics in dogs after an IT or an IF injection. Although this has not been investigated in this study, 30 min between the injection of local anaesthetic and clamping of the second testicle was an adequate time for both techniques to avoid nociceptive responses. Further research is warranted to investigate the spread of local anaesthetic when injected into the spermatic cord via IT and IF routes in dogs.

### 4.4 Limitations

This study has some limitations that need to be addressed. First, a lack of power calculation limits the interpretation of the results; the presence or absence of significant differences may not accurately reflect true effects. Further studies with a larger sample size are warranted to confirm the results presented here. Second, the study was not blinded, and the investigator who performed the intra and postoperative evaluations was aware of the group allocation. However, standardised cutoff values were used to reduce operator bias. Third, a non-echogenic needle was used to perform ultrasound-guided blocks, which may have rendered the block more difficult and time-consuming. However, needle and target visualisation were acceptable in all animals.

## 5 Conclusion

The UGIF block was a feasible alternative to the IT block, providing comparable analgesia. The time required to perform the UGIF was statistically, but not clinically, significantly different. The UGIF block seems to be an adequate alternative in dogs where an intratesticular injection is not recommended, such as in cases of testicular pathology.

## Data Availability

The raw data supporting the conclusions of this article will be made available by the authors, without undue reservation.
