# Correction: Comparison of analgesic efficacy between intrafunicular and intratesticular lidocaine injection in dogs

**DOI:** 10.3389/fvets.2026.1981226

**Published:** 2026-09-15

**Authors:** Frontiers Production Office

**Affiliations:** Frontiers Media SA, Lausanne, Switzerland

**Keywords:** dogs, intrafunicular, intratesticular, lidocaine, orchiectomy, regional anaesthesia, analgesia, cortisol

The funder University of Veterinary Medicine Vienna was erroneously omitted from the **Funding** statement. The correct Funding statement reads:

“The author(s) declared that financial support was received for this work and/or its publication. Open access funding provided by University of Veterinary Medicine Vienna.”

The original version of this article has been updated.

